# RIPK 1 in Alzheimer’s Disease: Research Progress Integrating Pathogenesis on Necroptosis-Related Neuroinflammation, and Potential Therapeutic Strategies

**DOI:** 10.3390/biomedicines14051155

**Published:** 2026-05-20

**Authors:** Ezgi Sila Toklucu, Shiqian Shen, Changning Wang, Can Zhang

**Affiliations:** 1 School of Medicine, Acibadem Mehmet Ali Aydinlar University, Atasehir, Istanbul 34752, Turkey; ezgi.toklucu@live.acibadem.edu.tr; 2 Genetics and Aging Research Unit, McCance Center for Brain Health, MassGeneral Institute for Neurodegenerative Disease, Department of Neurology, Massachusetts General Hospital, Harvard Medical School, Charlestown, MA 02129, USA; 3 Critical Care and Pain Medicine, Department of Anesthesia Massachusetts General Hospital, Harvard Medical School, Charlestown, MA 02129, USA; sshen2@mgh.harvard.edu; 4 Athinoula A. Martinos Center for Biomedical Imaging, Department of Radiology, Massachusetts General Hospital, Harvard Medical School, Charlestown, MA 02129, USA; cwang15@mgh.harvard.edu

**Keywords:** Alzheimer’s disease, RIPK1, necroptosis, amyloid-β, tau pathology, neuroinflammation, microglia, astrocytes, blood–brain barrier

## Abstract

**Background/Objectives**: Alzheimer’s disease (AD) is the most common cause of dementia worldwide; however, there is incomplete understanding of AD pathogenesis, and there are few disease-modifying treatments for AD. Research has begun to demonstrate that necroptosis, which is a regulated type of cell death mediated by receptor-interacting protein kinase 1 (RIPK1), plays a significant role in neurodegeneration and neuropathology associated with AD. The purpose of this review is to summarize current research regarding the role of RIPK1 in AD and possible therapeutic uses of RIPK1 inhibitors. **Methods**: This study is a narrative review of the literature summarizing experimental and clinical studies on RIPK1 signaling, necroptosis, neuroinflammation, and blood–brain barrier (BBB) dysfunction in AD. **Results**: The cumulative evidence demonstrates that RIPK1 activation associates with common AD pathways and particularly increases the severity of amyloid-β (Aβ) and tau pathology; promotes microglial activation; decreases the integrity of the BBB; and increases neuroinflammatory signaling in AD. Preclinical studies have shown that inhibiting RIPK1 genetically or pharmacologically in preclinical models decreased the extent of neurodegeneration and the rate of cognitive decline. **Conclusions**: RIPK1 is a key molecular link between necroptosis and neuroinflammation in AD. While the preclinical data are encouraging, further clinical research will be necessary to investigate RIPK1 changes in the brain, which may help better understand AD and establish the safety and effectiveness of potential therapeutic RIPK1 inhibitors in AD.

## 1. Introduction

Alzheimer’s disease (AD) is the most prevalent cause of dementia, and with people in most of the world growing older, it has become an urgent health issue. Today, there are an estimated 55 million people who live with dementia, and more than two-thirds are due to AD [1]. AD usually starts with subtle memory and cognitive deficits, gradually progresses to behavioral modification, and ultimately leads to loss of independence [2]. The illness is also marked by several key biological changes: tau proteins becoming hyperphosphorylated and spiraling up as neurofibrillary tangles inside neurons; accumulating amyloid plaques consisting of amyloid beta (Aβ) peptides with other proteins forming in the environment of neurons; uncontrolled neuroinflammation; progressive loss of neurons; and breakdown of synapses between cells [3,4,5]. While the exact cause is unknown and may differ among individuals, a combination of causes involving immune imbalance, vascular problems, environmental toxins, and genetic predisposition, especially the apolipoprotein E4 (*APOE4*) allele, seems to be responsible for the disease.

It has been found from longitudinal studies of people who have mutations causing AD in an autosomal dominant pattern that the abnormal brain pathology starts many years before any symptoms manifest clinically. The biomarker analysis indicates that there is deposition of Aβ and an increase in the neurofilament light chain, which appears to occur approximately 22 years before the appearance of symptoms, whereas the decrease in glucose metabolism occurs after 18 years, and atrophy occurs around 13 years before symptoms [6].

For several decades, researchers have assumed that neurodegeneration in AD is largely apoptosis-induced. That view was based on early signs such as caspase-3 activation, DNA fragmentation, and apoptotic bodies [7]. Yet closer pathological studies revealed a different finding: classical features of apoptosis are found rare in AD brains [8]. Postmortem tissue shows only low levels of apoptotic markers, while proteins linked to other forms of cell death appear in much higher amounts [9]. This suggests that apoptosis may explain only part of the picture and that other regulated cell death pathways are at work.

This is one of the newly identified pathways that includes necroptosis, which is an apoptosis-inhibited process of programmed necrosis mediated by receptor-interacting protein kinase 1 (RIPK1), receptor-interacting protein kinase 3 (RIPK3), and mixed lineage kinase domain-like protein (MLKL) [10]. The prevailing assumption about AD pathophysiology was that progressive neuron death was associated only with the process of apoptosis [11]. But new studies reveal another mode of programmed neurodegenerative cell death associated with necroptosis. Necroptosis differs from apoptosis because it leads to plasma membrane rupture and production of damage-associated molecular patterns (DAMPs). Unlike apoptosis, necroptosis causes inflammation that is characteristic of AD.

In addition to being increasingly appreciated for its involvement in the etiology of neurodegenerative disorders, RIPK1 is an evolutionarily highly conserved serine/threonine kinase that acts as an important regulator of inflammation and programmed cell death [12]. As an adaptor protein, it mediates signals received via different surface receptors, such as tumor necrosis factor receptor 1 (TNFR1) and pattern recognition receptors, including toll-like receptors, thus allowing for the coupling of inflammation- and stress-induced stimuli to their downstream effects on cellular signaling. Whether by virtue of specific modifications, such as ubiquitination, RIPK1 is able to mediate either the nuclear factor kappa B (NF-κB)-dependent pro-survival response or induce cell death, such as apoptosis and necroptosis. Thus, RIPK1 has been found to be involved in many more disorders than only those related to neurodegeneration including cancer, where it can act as both a tumor-promoting or tumor-suppressive factor depending on cellular context, and viral infections, where it participates in host–pathogen interactions by regulating inflammatory signaling and virus-induced cell death pathways [13].

In the context of neurodegeneration, RIPK1 functions as a central mediator of necroptosis, a multifunctional kinase which acts as a molecular switch, leading the cell to undergo necroptosis, apoptosis, or NF-κB-mediated survival signaling, depending on the specific cellular context [14,15]. Because of its central importance, RIPK1 appears particularly promising for AD studies. The growing body of literature confirms that RIPK1-dependent necroptosis is involved in AD pathogenesis: Aβ leads to necroptosis of both neuronal and glial cells, and RIPK1, RIPK3, and MLKL become highly activated in microglia due to Aβ oligomer exposure with a concomitant increase in cytokine secretion [16]. Additionally, several pieces of data from both animal experiments and human brain samples have provided the association between Aβ aggregation and RIPK1 activation [17]. Importantly, RIPK1 genetic or pharmacologic inhibition ameliorates Aβ-induced neurodegeneration and restores memory function [18].

The same story seems to apply to tau pathology. In vulnerable brain regions such as the hippocampus, hyperphosphorylated tau aggregates are often found alongside necroptosis markers [19]. It was shown that mutated tau can trigger RIPK1-dependent cell death. Related to that, silencing RIPK1 was seen to protect neurons [20]. More recently, when human neurons were transplanted into APP (amyloid protein precursor) knock-in mice, Aβ buildup worsened tau pathology while also enhancing RIPK1–RIPK3 signaling. A long non-coding RNA, maternally expressed gene 3 (MEG3), appeared to mediate this process [21]. These results collectively imply that tau pathology contributes to neurodegeneration by both accompanying and amplifying RIPK1 activity.

Neurons are not the only cells that undergo necrotic death; glial cells like astrocytes and microglia also play a significant role. These cells respond to Aβ and tau by releasing pro-inflammatory mediators, which in turn activate RIPK1 in adjacent neurons [22]. Necroptosis further triggers inflammation by stimulating microglia to release their contents into the tissue, which reinforces the inflammatory cycle. Microglia in models of AD also shift towards an extremely pro-inflammatory, also known as an M1-like, phenotype with enhanced RIPK1/RIPK3/MLKL activity [23]. Promisingly, RIPK1 inhibition can break this deadly spiral cycle, dampen inflammation, and drive microglia toward a more reparative, also known as M2-like, phenotype to enable Aβ clearance [24].

Vascular changes are also significant markers in Alzheimer’s brains and may make AD worse. Early in the disease, the blood–brain barrier (BBB) can become leaky, allowing blood-derived factors, including Aβ peptides and peripheral and immune cells from the blood to enter the brain and worsen inflammation [25]. Recent studies suggest that endothelial cells may undergo RIPK1-driven necroptosis, which can further weaken the BBB [26]. This type of endothelial cell death is associated with tight junction disruption and decreased pericyte support, according to analyses of postmortem Alzheimer’s brains [27]. Blocking RIPK1 can protect these cells, maintain the structure of brain blood vessels, and even enhance cognitive function, according to encouraging research conducted in animal models [28]. These findings demonstrate that RIPK1 is a crucial link between AD brain damage and vascular dysfunction.

**Figure 1:**
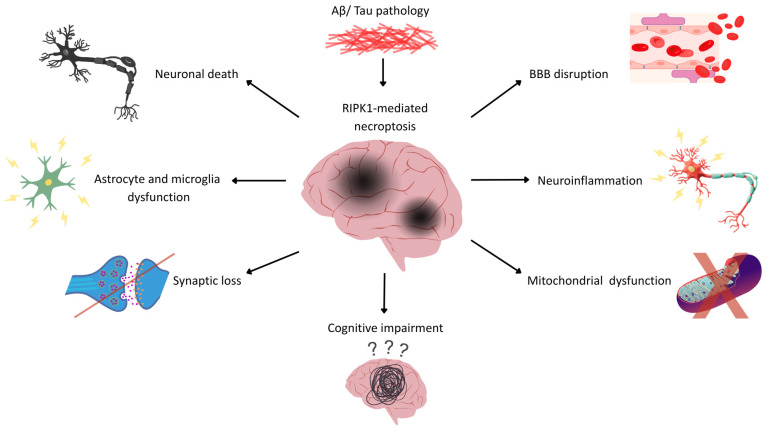
RIPK1-mediated necroptosis as a key contributor to AD pathology. In AD, RIPK1 engagement integrates the effects of Aβ and tau pathologies with mediated necroptotic cell death, which results in a series of events that drive AD pathology. RIPK1-mediated necroptosis contributes to neuronal death [3,4,19,32]; synapse loss [18,33]; mitochondrial dysfunction [19,34]; as well as astrocyte and microglial dysfunction that results in increased pro-inflammatory responses [7,16,20,22,35]; and necroptotic-mediated endothelial dysfunction that leads to BBB disruption [25,26,27,28,36]. All of this collectively serves to upregulate neuroinflammation [5,21,23,37], which culminates in cognitive impairment, the primary clinical outcome of AD [1,2]. Inhibiting RIPK1 has been shown in preclinical models to prevent neurodegeneration, protect synapses, promote vascular protection, and improve memory performance [18,28,33,38]. Together, this schematic emphasizes the role of RIPK1 as a critical molecular switch that acts to coordinate neuronal and glial cell death while linking inflammatory and vascular pathologies, suggesting that RIPK1 may be a viable target in AD. Created by the authors. Abbreviations: Aβ, amyloid beta; BBB, blood–brain barrier; RIPK1, receptor-interacting protein kinase 1.

Although the involvement of the RIPK1 pathway in AD is gaining more traction, it is unlikely to be applicable for every single clinical or pathologic subtype of this disorder. Currently, AD is recognized as a heterogeneous disorder with multiple subtypes characterized by distinct forms of cognitive impairment, neurodegeneration, biomarkers, and rates of progression. These include limbic-predominant, hippocampal-sparing, dysexecutive, posterior cortical atrophy, and logopenic variants, where the degree of tau accumulation, inflammation, cerebrovascular alterations, or microglial activation might play a differential role in RIPK1-mediated signaling [29]. In addition, biomarker-defined subtypes with different APOE genotype, inflammation, or cerebrovascular features may vary in terms of their susceptibility to necroptosis. APOE4-positive AD patients, for example, display higher BBB impairment and microglia activation, processes heavily related to RIPK1 signaling. The same applies to rapidly progressing or atypical AD phenotypes, whose inflammatory and necroptotic mechanisms may be enhanced in comparison to slowly progressive amnestic subtypes. In light of this, future research should focus on RIPK1 signaling pathways in clinicopathological subtypes of AD rather than consider the disease homogenous in nature [30]. Taken together, Aβ plaque accumulation, tau tangles, glial activation, chronic inflammation, and BBB breakdown are the core pathological features of AD, and they are increasingly associated with RIPK1-mediated necroptosis. This mechanism offers a way to bring together many of the changes seen in AD. Notably, consistent with biochemical findings, molecular probes of RIPK1 have been reported, which precisely visualize RIPK1 in the brains of Alzheimer’s transgenic mice, providing useful tools to “see” RIPK1 spatial–temporal changes with AD progression [31]. Also, RIPK1 is a key mediator of inflammation and neurodegeneration, as well as a switch for cell death. RIPK1 plays an essential role in the brain by altering tau and Aβ metabolism, regulating glial cell functions, and driving microvascular damage in the cerebral vasculature. Discovering the role of RIPK1 in different pathways and its diverse effects not only contributes to understanding the concept of neurodegeneration and neurodegenerative diseases like AD, but it also reveals its potential to become a biomarker and therapeutic target. Thus, the characterization of RIPK1’s role in AD pathology could be a milestone to comprehend the complex pathways and interconnected processes that might be the trigger in the onset of this debilitating disease. Taken together, RIPK1 emerges as a central hub linking amyloid and tau pathology, neuroinflammation, and vascular dysfunction in AD, as shown in Figure 1.

In this narrative review, we integrate current experimental and translational evidence to discuss how RIPK1 links necroptosis, neuroinflammation, and vascular dysfunction in AD.

### 1.1. Literature Search Strategy

This article is a narrative review of the literature and does not follow a systematic review or meta-analysis protocol. The literature review was done to find studies examining RIPK1’s role in AD from both a basic and translational perspective. The literature review utilized searches of three major scientific databases (PubMed, Web of Science, and Scopus) for all publications available through December 2025. In addition, to locate studies that would be included in this review, the reference lists of selected studies were reviewed by hand. The following keywords were searched as well as their combinations: “tau,” “amyloid-β,” “neuroinflammation,” “microglia,” “blood-brain barrier,” “necroptosis,” “Alzheimer’s disease,” and “RIPK1.”

### 1.2. Eligibility and Study Selection Requirements

This review included peer-reviewed original research articles and relevant review papers published in English. Studies were considered eligible if they examined RIPK1 signaling in the context of AD-related pathological processes, such as neuronal degeneration, glial activation, neuroinflammatory responses, or cerebrovascular dysfunction. The evidence collected included findings that came from analyses of brain tissues, in vitro and in vivo experimental models. All the studies that were strictly related to other neurological diseases and cell death processes independent of RIPK1 were not taken into consideration. More attention was paid to the studies where there is some kind of mechanistic explanation, especially regarding the role of RIPK1 in AD.

## 2. RIPK1 and Aβ Pathology

Aβ accumulation and formation into oligomers and hallmark amyloid plaques are central to AD. Recent studies recognize Aβ as an initiator that activates RIPK1 and necroptosis, promoting neurodegeneration. It established that Aβ oligomers induce necroptotic death in AD models via microglial activation. Microglial exposure to Aβ activated RIPK1, RIPK3, and MLKL phosphorylation, and pharmacologic inhibition of RIPK1 or MLKL profoundly reduced cytokine secretion and neuronal loss [34,39,40]. Similarly, AD patient brain tissue shows elevated Aβ burden associated with RIPK1/RIPK3 activation.

Incidentally, RIPK1 is also downstream of inflammatory stimuli. Tumor necrosis factor-α (TNF-α), potentially released by Aβ-activated microglia, robustly induces RIPK1-dependent necroptosis. Another study indicates that oxidative stress due to the effect of Aβ contributes to mitochondrial DNA (mtDNA) oxidation and cleavage and the conversion of mtDNA from B-form to Z-DNA structure recognized by ZBP1, resulting in the development of neuroinflammation mediated via RIPK1 independently of cell death [41]. Injection of TNF-α into brain tissues leads to significant activation and neurodegeneration in mouse models for both wild-type mice and AD [39,40]. In agreement with this, RIPK1 kinase activity-deficient mice or necroptosis inhibitor-treated animals have reduced Aβ-induced pathology and improved maze test learning [40]. Thus, the amyloid cascade in AD would appear to include RIPK1-dependent necroptosis as a downstream event of neuronal injury.

**Figure 2:**
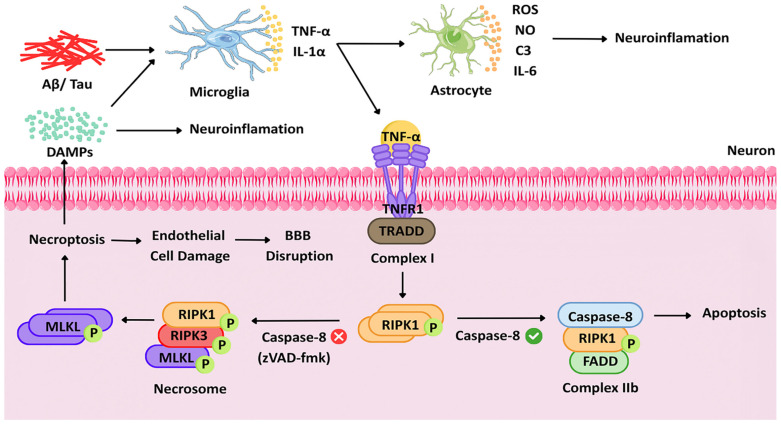
RIPK1 as a central regulator of apoptosis and necroptosis downstream of TNF-α/TNFR1 signaling in neuroinflammation. In AD, Aβ plaques and tau tangles pathologically associate with stimulated microglia, which release pro-inflammatory cytokines such as TNF-α and IL-1α [5,42]. These cytokines induce astrocytes and the release of reactive oxygen species (ROS), NO, C3, and IL-6. Together, these factors amplify neuroinflammation, promote endothelial cell injury [37], and contribute to BBB disruption [23]. In parallel, damage-associated molecular patterns (DAMPs) and TNF-α activate TNFR1 signaling [37], leading to the recruitment of TRADD and the assembly of Complex I at the plasma membrane [39]. This Complex I is presented in a simplified schematic form and promotes RIPK1 activation involving TRADD, TRAF2/5, and cIAP1/2, leading to RIPK1 ubiquitination/phosphorylation and the initiation of NF-κB-dependent pro-survival and inflammatory signaling, which then acts as a signaling hub to determine cell fate [43]. Particularly, when caspase-8 is inhibited by benzyloxycarbonyl-Val-Ala-Asp-fluoromethylketone (zVAD-fmk) [44], RIPK1 associates with RIPK3 to form the necrosome, resulting in MLKL phosphorylation and necroptosis. In AD, necroptosis occurs in neurons of hippocampal regions showing elevated phosphorylated RIPK3 (p-RIPK3) and phosphorylated MLKL (p-MLKL) [22]. In contrast, when caspase-8 is active, RIPK1 joins with Fas-associated protein with death domain (FADD) and caspase-8 to form Complex IIb, driving apoptosis [37]. RIPK3, as part of the necrosome, amplifies DAMP release, further increasing microglia responses through a vicious cycle of DAMP-mediated microglial activation [42]. Thus, RIPK1 integrates inflammatory and death signals to link protein aggregation, glial activation, and cytokine release to neuronal cell death during neuroinflammation. Created by the authors. Abbreviations: AD, Alzheimer’s Disease; Aβ, amyloid beta; TNF-α, tumor necrosis factor alpha; IL-1α, interleukin-1 alpha; ROS, reactive oxygen species; NO, nitric oxide; C3, complement component 3; IL-6, interleukin-6; DAMPs, damage-associated molecular patterns; BBB, blood–brain barrier; TNFR1, tumor necrosis factor receptor 1; TRADD, TNFR1-associated death domain protein; TRAF2/5, tumor necrosis factor receptor-associated factor 2 and 5; cIAP1/2, cellular inhibitor of apoptosis protein 1 and 2; NF-κB, nuclear factor kappa B; caspase-8, cysteinyl aspartate-specific protease 8; RIPK1, receptor-interacting protein kinase 1; RIPK3, receptor-interacting protein kinase 3; MLKL, mixed lineage kinase domain-like protein; FADD, Fas-associated protein with death domain; zVAD-fmk, z-Val-Ala-Asp-fluoromethylketone.

In terms of the RIPK1 signaling complex, Aβ deposits and tau deposition cause microglia activation and release of pro-inflammatory cytokines such as TNF-α and IL-1α [5,42]. In the case of activation of TNFR1, RIPK1 binds TRADD, TRAF2/5, and cIAP1/2 to initiate Complex I, where activation of NF-κB leads to inflammatory response and pro-survival signaling [39,43]. In case cysteinyl aspartate-specific protease 8 (caspase-8) is inhibited, then the binding of RIPK1 and RIPK3 forms necrosome and MLKL phosphorylation occurs for necroptosis, while on the other hand, activation of caspase-8 causes complex IIb formation along with FADD, leading to apoptosis [22,44]. Complex IIa is only an intermediate that may form in some cases [43]; however, attention in this figure is drawn to Complex IIb and necrosome, two downstream complexes involved in causing the activation of the pathway resulting in inflammatory cell death. This feed-forward loop linking Aβ, TNF-α release, and RIPK1-driven necroptosis is summarized in Figure 2.

RIPK1, in the pathogenesis of AD, is induced by inflammatory signals resulting from Aβ, and its involvement in disease progression is mainly based on Aβ clearance and not on Aβ synthesis. According to experimental evidence, inhibition of RIPK1 either genetically or pharmacologically results in the reduction in Aβ load in vivo, indicating an indirect but physiologically significant action in the regulation of Aβ pathology. The mechanism involves RIPK1-mediated regulation of microglia phenotypes, resulting in the promotion of the inflammatory phenotype with reduced ability of microglia to phagocyte Aβ. The inhibition of RIPK1 results in microglia with a more homeostatic and phagocytic phenotype, thus facilitating Aβ clearance and limiting its accumulation [45].

## 3. RIPK1 and Tau Pathology

Intracellular tau-associated neurofibrillary tangles usually occur downstream of amyloid pathology and correlate more robustly than amyloid pathology with neuronal loss in AD, and new evidence points towards the involvement of RIPK1 in tauopathy. Histopathological analyses of AD brains reveal that clusters of hyperphosphorylated tau often colocalize with markers of activated necroptosis. Phosphorylated RIPK1/3 and MLKL accumulated in hippocampal neuron granuovacuolar degeneration bodies (GVD), along with tau inclusions [46]. In vitro, mutant tau (P301S) or tau oligomer overexpression induced RIPK1-dependent necroptosis; genetic or pharmacologic suppression of RIPK1 kinase activity rescued neurons against tau-induced death. A breakthrough report recently grafted human neurons into an APP knock-in mouse: Aβ deposition triggered tau pathology and a spectacular induction of neuronal RIPK1-RIPK3 signaling by the long noncoding RNA MEG3. MEG3 knockdown in these human neurons reduced RIPK1/MLKL activation and cell death, indicating a tau-dependent, RIPK1-mediated death pathway [16].

Taken together, these findings suggest that tau pathology may drive an increase in RIPK1 signaling. In AD patients, neurofibrillary tangle burden correlated with necroptosis markers. In addition, RIPK1 kinase activity may create a self-reinforcing vicious cycle: activating RIPK1 by driving inflammatory caspase activation and tau phosphorylation, which drives subsequent necrosome formation. Significantly, GVD, a feature of AD, is now shown to be a morphological marker of neuronal RIPK1-mediated necroptosis [16,46].

## 4. RIPK1 and Neuroinflammation

RIPK1 is also a key switch between cell death and inflammatory signaling. Activated glia and chronic neuroinflammation exacerbate pathology in the AD brain, and RIPK1 has been directly implicated in the immune dysregulations. Aβ- and tau-exposed microglia and astrocytes secrete TNF-α and other cytokines that can induce RIPK1 activation in nearby neurons and glia. Necroptosis of microglia or astrocytes themselves can instead exacerbate neuroinflammation by extruding intracellular contents. It is discovered that necroptosis triggered by Aβ in cultured microglia and monocytes led to the release of pro-inflammatory mediators, creating a feed-forward cycle of injury [7].

Experimental models illustrate the cycle. In AD-model mice, subsets of microglia acquire a neurotoxic “M1” phenotype with elevated RIPK3 and MLKL activation. The inhibition of RIPK3 or MLKL polarizes microglia towards an anti-inflammatory M2 phenotype, suppressing cytokine production and facilitating Aβ clearance. Along similar lines, increased RIPK1 kinase activity was found in AD brain microglia, and pharmacologic inhibition of RIPK1 decreased M1 microglia and inflammatory gene expression [35]. These studies highlight RIPK1’s commanding position in microglial biology: its activation imposes a pro-inflammatory program that worsens AD pathology, while its inhibition alleviates neuroinflammation.

Outside of microglia, RIPK1 signals intersect with other innate immune pathways. For example, receptor-interacting serine/threonine protein (RIP) kinases activate the NF-κB pathway and chronic TNF signaling (augmented in AD) permits RIPK1 activation [21,39]. Necroptosis is inflammatory in itself: membrane destruction by phospho-MLKL releases DAMPs that also activate astrocytes and microglia, linking cell death with inflammation [37]. Consequently, RIPK1 is at the nexus of inflammation and cell death in AD, and its hyperactivation contributes causally to the vicious circle of chronic neuroinflammation and progressive neuronal injury.

Aβ and tau pathology become chronic, triggering the activation of glial cells (microglia and astrocytes), which leads to the secretion of TNF-α. The secretion of TNF-α (along with other stimuli) leads to the activation of the RIPK1 complex in neurons and endothelial cells, leading to the recruitment of RIPK3 and phosphorylation of MLKL. Phosphorylated MLKL triggers necroptosis and cytokine secretion, resulting in neuroinflammation. Necroptosis triggered by RIPK1 is also activated in endothelial cells (leading to BBB dysfunction) and microglia (promoting inflammation) [5].

## 5. Neuronal Necroptosis and Regulated Cell Death

Neurodegeneration is the main and direct indicator of cognitive dysfunction in AD. In contrast to classical apoptosis that relies almost exclusively on caspase activation and is “silent,” necroptosis is a process of cell lysis and inflammation. AD neurons possess certain morphological and biochemical hallmarks of necroptosis. High levels of phosphorylation or activation of RIPK1, RIPK3, and MLKL have been found in neurons of damaged areas (e.g., the hippocampus) [19,22]. Importantly, studies demonstrate the presence of the specific active complex necrosome exclusively in neurons: while the selectivity of phosphorylated RIPK1/phosphorylated RIPK3 (p-RIPK1/p-RIPK3) expression was detected in the neurons of the AD cortex, no signs of its existence were seen in any non-neural cells [46]. AD brain in vivo and AD brain in vitro showed evidence of necroptosis but not apoptosis (swelling of cells, permeabilization of membranes) [4].

Inactivation of the gene encoding RIPK1 (mutant) and RIPK3 knockout (KO) in APP/PS1 (Presenilin-1) mouse models preserved neuronal numbers and inhibited behavioral changes [21]. At the same time, application of RIPK1 inhibitors (e.g., necrostatin-1) prevented neuron loss caused by Aβ/tau pathology and memory impairment in the AD mouse brain [18]. These therapeutic interventions decreased neurodegeneration and synaptic injury in the hippocampus and proved to be an crucial central cell-death effector in AD for necroptosis.

Metabolic modulators, including kinases and hydroxylases, have been known to regulate apoptosis by modulating the level of RIPK1. For example, AMPK phosphorylates RIPK1 at the serine residues S415/S416, thereby giving another metabolic control point for regulating cell death and the resulting inflammatory response under conditions of energy deprivation. Conversely, failure to phosphorylate RIPK1 during energy stress leads to apoptosis induction by RIPK1 and tissue injury, especially in ischemic conditions [47]. Under normoxic conditions, EGLN1-mediated proline hydroxylation of RIPK1 at P195 is recognized by pVHL to inhibit the kinase activity in a tonic manner. Prolonged periods of hypoxia or the absence of this inhibitory mechanism result in unrestricted activation of RIPK1 followed by necroptosis and inflammation, thus leading to the ischemic disease states [48].

Necroptosis is a specific type of programmed cell death that does not utilize apoptotic processes. In Alzheimer’s patients, usual indicators of apoptosis such as cleaved caspase-3 and DNA laddering are not common, while necroptosis-related markers occur abundantly following prolonged disease development. Another experiment indicates that neuron cell death in Alzheimer’s disease is mediated by RIPK1-dependent necroptosis triggered by lncRNAs, with the formation of active necrosomes inside GVD bodies [39]. Therefore, neuronal cell death in Alzheimer’s disease involves abundant RIPK1-dependent necroptosis [32,39].

## 6. RIPK1 in Cerebrovascular Dysfunction and BBB Integrity

Cerebrovascular damage and BBB disruption emerge as early characteristics of AD. Recent studies point toward RIPK1 aggravating endothelial damage and causing chronic damage in organs [49]. RIPK1-mediated necroptosis has been observed in brain endothelial cells in AD models and patients [36]. AD-model mice were found to exhibit reduced expression of the acetyltransferases mNat1/hNAT2 in endothelium, leading to consequent spontaneous activation of RIPK1/RIPK3/MLKL and death of endothelial cells [23]. Pharmacological inhibition of RIPK1 in mice preserved endothelial cell survival, increased microvascular integrity, and reduced cognitive impairment [36].

Activated endothelial RIPK1 was correlated with focal BBB leakage. Endothelial necroptosis in human AD tissue was correlated with the loss of tight junction proteins and pericyte loss [50]. Interestingly, pharmacological inhibition of RIPK1 in models of brain injury (e.g., ischemia) has been shown to reduce BBB permeability [51,52]. Thus, there is evidence that AD-related cerebrovascular dysfunction is, at least in part, mediated by RIPK1-dependent necroptosis of endothelial cells.

BBB disruption in AD causes peripheral blood-derived factors, including Aβ peptides and peripheral immune cells to go into the brain, and these toxins cause exacerbated neuroinflammation reactions. By mediating selective endothelial loss, RIPK1 activation is bound to promote such initial pathology. It was shown that RIPK1 kinase-inactive transgenic models (D138N and S161N) had reduced transepithelial electrical resistance (TEER) loss and sodium fluorescein (NaFl) leakage through the BBB [24]. Nec-1 can stabilize the BBB after a TNF-α insult or stroke and can be used as a therapeutic strategy that way [38]. These mechanisms align with the schematic shown in Figure 2, where MLKL-driven necroptosis contributes to endothelial injury and BBB disruption. These findings make RIPK1 an important factor as a connection between AD vascular and parenchymal pathology: endothelial necroptosis disrupts BBB integrity, perhaps driving Aβ and tau deposition.

In light of recent research, it is clear that alterations in the role of the BBB in the neurodegenerative processes can be viewed in connection with cerebral blood flow rather than an isolated vascular process. For instance, in a condition known as corticobasal syndrome, which is caused by the accumulation of tau protein, there have been cases reported of altered cerebral blood flow in conjunction with blood-related inflammatory factors such as the neutrophil-to-high density lipoprotein ratio [53].

## 7. Molecular Regulation of RIPK1 and Changes in Proinflammatory Cytokine

RIPK1 activity is stringently regulated by upstream signals and post-translational modifications. Knowledge of these processes is essential to linking RIPK1 with AD. The most well-characterized upstream regulator is TNF-α, which induces signaling upon interaction with tumor necrosis factor receptor 1 (TNFR1) [43]. Under normal conditions, TNF-TNFR1 signaling creates a membrane-bound complex I (TRADD, TRAF2, RIPK1, etc.), which is pro-survival and NF-κB activating. But when Complex I is compromised (e.g., by deubiquitination of RIPK1 or inhibition of transforming growth factor beta-activated kinase 1 (TAK1)), RIPK1 becomes phosphorylated (S166) and leaves to create a cytosolic death complex [39].

In AD, TNF is elevated, and TNFR1 signaling can become biased towards necroptosis. Other death receptors (Fas/CD95) and pattern receptors such as toll-like receptor 4 (TLR4) also stimulate RIPK1/RIPK3, linking viral or damage signals to necroptosis [54,55]. Genetic modifiers also regulate RIPK1 control: e.g., a novel SHANK-associated RH domain interactor (SHARPIN) variant has been found to be associated with late-onset AD [56]. SHARPIN is part of the linear ubiquitin chain assembly (LUBAC) complex that conjugates linear ubiquitin chains to RIPK1; the AD-associated mutation destabilizes RIPK1 ubiquitination, thus predisposing it to kinase activation. TAK1 phosphorylates the intermediate domain of RIPK1 on stimulation by TNFα, thereby modulating cell death mechanisms: transient phosphorylation leads to RIPK1-independent apoptosis, loss of phosphorylation enables FADD binding to RIPK1 and causes RIPK1-dependent apoptosis (RDA), while sustained phosphorylation triggers RIPK1–RIPK3 interaction and necroptosis [57]. Although detailed analyses of TAK1 in AD are lacking, it is possible that aberrant TAK1 signaling could contribute to RIPK1-dependent pathogenesis.

Post-translational modifications regulate the fate of RIPK1. These include phosphorylation, which plays an essential role, as evidenced by phosphorylation at S166 in the kinase domain of RIPK1, which is considered an indication of its activation. Increased levels of pS166 RIPK1 in AD brains and models have been reported by multiple research teams [21]. In downstream events, RIPK3 becomes phosphorylated [13] and phosphorylates MLKL, resulting in cellular membrane disruption [10]. Both RIPK1 and RIPK3 possess receptor-interacting protein homotypic interaction motif (RHIM) domains for their binding [58]. Additionally, ubiquitination of RIPK1 by cIAPs and LUBAC complex is vital, as mediated by the LUBAC components of Complex I (HOIP/HOIL-1/SHARPIN) [59]. These ubiquitin chains recruit prosurvival factors and inhibit RIPK1’s kinase activity. In AD, several studies report decreased levels of cIAP1/2 and increased deubiquitinase CYLD [34], which would remove ubiquitin and allow activation of RIPK1. A switch towards RIPK1 deubiquitination (by loss of SHARPIN or overexpression of CYLD) promotes necroptosis. Together, these post-translational modifications (PTMs) form a switch: M1/K63 ubiquitination and non-phosphorylated RIPK1 promote NF-κB survival, and deubiquitination and S166 phosphorylation promote necrosome formation.

The interaction between RIPK1 and RIPK3 occurs in necrosome (RIPK1–RIPK3–MLKL complex) using the RHIM domains [60]. The result is autophosphorylation of RIPK3 and its conformational change, enabling phosphorylation of MLKL [61]. Subsequently, phospho-MLKL oligomerizes and localizes on membranes, forming pores that ultimately cause cell death [62]. In AD, deletion of either RIPK3 or MLKL works as efficiently as inhibiting RIPK1: for example, knockout of RIPK3 and expression of kinase dead MLKL in mice causes lesser neuronal loss and inflammation by selectively blocking necroptotic cell death [63]. On this basis, some RIPK3 inhibitors, including dabrafenib and ponatinib, inhibit MLKL activation and proved useful in xenografts of AD [39].

RIPK1 signaling exhibits cell-type specificity. RIPK1 is controlled in neurons mostly by FLIP/Caspase-8 under normal conditions, but under chronic insult, neurons readily succumb to necroptosis [64]. In microglia, the kinase activity of RIPK3 has been shown to induce an inflammatory M1 type [35]; interestingly, activation of necrosomes in microglia can induce cytokine release without necessarily leading to immediate cell death, indicative of a dual function in survival versus cell death. Astrocytes could also undergo RIPK1-mediated death under AD-like conditions, although evidence is limited. Overall, neuronal RIPK1 activation leads to self-destruction; microglial/endothelial RIPK1 activation is linked to glial dysfunction and BBB breakdown, all of which serves to perpetuate AD pathology [5].

Proinflammatory cytokines and chemokines regulated by the RIPK1 pathway in AD include several members of the interleukin (IL) family. Among them, IL-18 is increased together with TNF-α in necroptotic neurons [11,49]. IL-33 is released as a necroptotic DAMP in RIPK1-deficient mice [63], while roles for IL-36 and IL-37 in necroptosis-mediated inflammation remain to be identified [11]. IL-15 is increased in the phosphorylated tau (pTau)-expressing neuronal models, where it acts through the RIPK1/RIPK3/MLKL pathway [18]. IL-12 and IL-2, on the other hand, are found to be downregulated when RIPK1 is inhibited in mouse models of neurodegeneration [17,35]. IL-8, on the contrary, is induced by upstream regulator macrophage migration inhibitory factor (MIF) [52].

The necrosome also induces chemokines CCL2 (C–C motif chemokine ligand 2), CCL5, CXCL9 (C–X–C motif chemokine ligand), and CXCL10 in phosphorylated Tau (pTau)-stimulated neurons, wherein CCL5 and CXCL9 are even stronger inducers than TNF-α and IL-6 [17]. CCL3 and CCL4 are elevated in LPS-induced inflammation but reduced upon inhibition of RIPK1. Type I interferons (IFN-α4, IFN-β1) and tumor necrosis factor-related apoptosis-inducing ligands (TRAIL) (TNFSF10) are induced through the RIPK1/RIPK3/MLKL pathway [54]. RIPK1 also controls other inflammatory mediators like Cyclophilin A, which is increased in AD patients’ brain endothelium and pericytes, and CSF-2 (Granulocyte–Macrophage Colony-Stimulating Factor), which is dramatically reduced when RIPK1 kinase activity is inhibited [27,54]. Notably, necroptosis inhibition, either by RIPK3 deletion or MLKL, polarizes the immune system to anti-inflammatory cytokines such as IL-4 and IL-10, demonstrating that RIPK1 is critical in balancing pro- and anti-inflammatory signaling in AD [35].

## 8. RIPK1 in Other Neurodegenerative Diseases

RIPK1 is not specific to Alzheimer’s disease and has been implicated in several other neurodegenerative disorders characterized by chronic neuroinflammation and regulated cell death.

In amyotrophic lateral sclerosis (ALS), RIPK1 contributes to neuroinflammatory signaling and necroptosis, promoting neuronal and glial dysfunction. Pharmacological or genetic inhibition of RIPK1 has been shown in preclinical models to reduce inflammatory responses and partially modify disease progression, supporting its functional involvement in ALS pathology [16]. In multiple sclerosis (MS), RIPK1 is involved in oligodendrocyte cell death and demyelination processes [65]. Experimental autoimmune encephalomyelitis (EAE) models further support this role, where RIPK1 inhibition has been associated with reduced neuroinflammation and attenuated disease severity [66]. In Parkinson’s disease (PD), emerging evidence suggests that RIPK1 may contribute to dopaminergic neuron vulnerability through inflammation-associated and cellular stress-related pathways, although its precise mechanistic role remains less well defined compared to ALS and MS [67].

Additionally, increased RIPK1 activity has been reported in tauopathies, including frontotemporal dementia (FTD) and progressive supranuclear palsy (PSP). In these disorders, RIPK1 is associated with sustained microglial and astrocytic activation, contributing to chronic neuroinflammatory states [68]. Overall, RIPK1 appears to function as a shared upstream regulator of neuroinflammatory and regulated cell death pathways across multiple neurodegenerative diseases, rather than acting as a disease-specific driver.

## 9. Translational Relevance

Prior to delving into RIPK1-targeted biomarkers and treatments, one should review the wider scope of therapies for AD.

The success of AD therapy relies not only on how much brain amyloid can be removed but also on proper patient selection. Studies indicate that lowering brain amyloid below a critical level (<24 centiloids), especially in the early stages of disease onset, might be required to observe clinical benefits. The most favorable results can be achieved in people who have a “Goldilocks” level of tau protein [69].

The therapy landscape for AD has changed significantly in recent years, with many drugs having received approval after a relatively long period during which no significant progress was made. Modern drugs are targeting novel mechanisms of action such as neuroinflammation, immune system components, and tau proteins rather than amyloid alone. It is also reflected in the current trends within the industry, where small firms are usually responsible for initiating research projects, whereas large companies carry out their development [70].

### 9.1. RIPK1 as a Biomarker

RIPK1 (and its activated status) is also being explored as a biomarker because it occupies critical mechanisms in AD. Postmortem research indicates that elevated RIPK1 and MLKL levels correlate with lower cognitive scores in patients with AD [71]. For instance, RIPK1 expression in the brain correlates inversely with brain weight, suggesting that burden of necroptosis may be used as a marker of disease severity [32]. Similarly, in APP/PS1 mice, markers of necroptosis are associated with behavioral impairment. Oral RIPK1 inhibitor DNL747 was found to lower markedly p-RIPK1^S166^ in peripheral blood mononuclear cells (PBMCs) of patients, confirming on-target effects [72]. Whether these also hold true for central nervous system (CNS) activity is being explored. Genetic mutations like the SHARPIN variant also imply personal susceptibility to RIPK1-mediated pathology, as it utilizes the NF-κB pathway for the regulation of immunologic and inflammatory responses [73].

Future studies could focus on developing PET tracers to visualize necroptosis or using CSF (cerebrospinal fluid) proteomics to track RIPK1 and MLKL peptides as dynamic biomarkers. RIPK1 signaling may also have implications not only in established AD but also in its prodromal forms such as mild cognitive impairment (MCI), which often precedes AD dementia and is especially common among biomarker-positive amnestic MCI. Inflammation, breakdown of the BBB, and synapse damage have been identified in individuals with MCI, raising the possibility that RIPK1-related processes can start getting impaired even before neurodegeneration becomes evident. Given that RIPK1 combines elements of inflammation, neurodegeneration, and BBB breakdown, tracking the status of RIPK1 during MCI may be useful in predicting who is more likely to develop AD. Indeed, treating patients at prodromal rather than advanced stages of the disease may have more benefits than treating patients with AD due to irreversible damage, especially if treatment targets RIPK1 itself. Nevertheless, there is insufficient data on the role of RIPK1 in MCI specifically [15,74].

An overview of RIPK1-related biomarkers currently under investigation is provided in Table 1. RIPK1 has been found to be involved in the process of GVD-necroptosis, which occurs when the necrosome assembly takes place inside the granulovacuolar degeneration vesicles when there are p-tau tangles and not Aβ pathogenesis alone. Treating these pathways using drugs that can enter the brain such as ponatinib leads to inhibition of downstream targets [75].

### 9.2. Therapeutic Targeting and Strategies

Preclinical studies show that both genetic and pharmacologic inhibition of RIPK1 is protective in mouse models of AD. Mice lacking kinase-active RIPK1 (RIPK1^D138N^) or RIPK3- or MLKL-deficient mice lose fewer synapses and perform better on memory tests [33]. Small molecules like necrostatin-1 (Nec-1, RIPK1 inhibitor) or necrostatin-1 stable analog (Nec-1s) and MLKL inhibitors improve neuroinflammation and neuronal loss in AD models [38]. RIPK1 inhibition also inhibits glial activation and BBB leakage in these models. Interestingly, re-purposed kinase inhibitors like ponatinib, dabrafenib, and sorafenib that suppress RIPK3 have proven useful in AD xenograft models [39]. These observations indicate RIPK1 as a promising target in preclinical AD.

In a different preclinical model of tauopathies, scientists observed elevated levels of RIPK1 in brain tissue in both animal models and human patients with frontotemporal dementia, progressive supranuclear palsy, and AD. Thus, an experiment was conducted to determine the efficacy of an RIPK1 inhibitor, GSK2982772 [68]. It was observed that while the treatment strongly inhibited neuroinflammatory markers, i.e., the astrocyte reaction response, it did not prevent progressive neurodegeneration in the hippocampal region. This observation demonstrates that inhibition of RIPK1 will cure neuroinflammation but is ineffective as a monotherapy against neuronal loss in these illnesses.

Several RIPK1 inhibitors reached human trials in neurodegenerative diseases, a major translational advance from preclinical activity to clinical application. Denali Therapeutics initially identified DNL747 (SAR443060), the first CNS-permeating RIPK1 inhibitor ever given in AD. Early Phase I trials showed that DNL747 was generally well tolerated, crossed CSF sufficiency, and engaged the target as indicated by reduced p-RIPK1 levels in peripheral blood mononuclear cells [72]. Despite these promising results, progress was severely hampered by issues of pharmacokinetics (PK), and long-term toxicology screens revealed unforeseen issues of safety that led to program closure. In response to these problems, Denali and Sanofi subsequently advanced DNL788 (SAR443820), a second-generation RIPK1 inhibitor possessing improved PK and CNS penetration.

At 50–100 mg in a Phase I trial, DNL788 was safe and well tolerated among healthy volunteers with demonstration of peripheral target engagement [78]. Subsequent to this, the compound has progressed into Phase II trials in ALS and MS, receiving FDA Fast Track designation in ALS, and an exploratory study in AD is on the horizon. This lesson emphasizes the strategy of optimizing RIPK1 inhibitors in order to overcome preliminary safety and distribution issues. Other companies are concurrently involved in active process development for second-generation RIPK1 inhibitors.

In addition to these compounds, emerging RIPK1 inhibitors such as GFH312 are currently under early clinical evaluation. GFH312 is a highly selective RIPK1 inhibitor that has completed Phase I and is planned to be evaluated in Phase II trials. Preclinical studies have shown that GFH312 effectively blocks necroptosis and offers neuroprotection but was discontinued due to adjustments in clinical development [76].

Sironax SIR-2446 is an oral and CNS-penetrating molecule in Phase I trials for AD and MS. Preliminary evidence suggests that it is safe, well tolerated, and capable of delivering potent target inhibition within peripheral biomarkers [79]. Apart from these direct AD programs, other RIPK1 inhibitors are under development primarily for systemic inflammatory diseases, such as Genentech’s GDC-8264 and Lilly’s LY3871801, although their mechanisms may give some clues to possible uses in neurodegeneration. Combination strategies are also being envisioned: given that necroptosis is involved in amyloid and tau pathology, RIPK1 inhibition may synergize with existing anti-amyloid or anti-tau treatments, possibly prolonging their therapeutic benefit.

Despite the progress, it is noteworthy that to date, no RIPK1 inhibitor has demonstrated clinical effectiveness in AD patients. It was reported that DNL747 advanced to a Phase II trial but was subsequently discontinued based on toxicology findings [72]. One of the most progressed CNS-penetrant candidates is DNL788, but its clinical effectiveness in neurodegeneration is yet to be established.

Supported by recent successes of Alzheimer’s drug discovery bringing Aβ-reducing antibodies as disease-modifying treatments for AD, which has been made possible by precisely testing amyloid pathology by molecular imaging, probes for RIPK1 may help the future path of developing new RIPK1 inhibitors. Together, many studies suggest the potential promise of inhibiting RIPK1 in AD: it may be possible pharmacologically to inhibit RIPK1 action without adverse effects, but safety optimization, penetration into the CNS, patient stratification, and therapeutic effect evaluation will be crucial to ultimate research success. Collectively, these advances highlight the translational potential of targeting RIPK1 in AD, with key preclinical and clinical studies summarized in Table 1.

## 10. Limitations and Setbacks

Unfortunately, clinical and preclinical studies that target RIPK1 have different limitations and hardships that slow down the progression of current studies, cause discontinuations in clinical trials, and prevent the initiation of new trials. Limitations can be grouped under categories of discontinuation and safety concerns, insufficient access to the target site, sampling limitations, translational gaps, selection bias, statistical problems, and experimental/technical constraints, as shown in Table 2.

Especially for clinical trials, the biggest progression barrier is safety and tolerability. Serious adverse effects caused withdrawals of trials from development. Those adverse effects include immune-mediated anemia and thrombocytopenia observed in nonhuman primates; hepatotoxicity shown by increased ALT/AST in healthy participants; and morbilliform rash in higher dose cohorts [72,78,79]. Another cause is efficacy insufficiency. The DNL788 trial did not manage to show the expected therapeutic effect in the Phase 2 trial on patients with ALS [39]. Strategic reasons were presented for withdrawal of GFH312 in Phase 2 trials before recruitment [76].

The other significant problem faced is poor central nervous system (CNS) penetration and the inability to directly assess target engagement in disease sites. For instance, GSK2982772 had poor CNS penetration due to its ability to undergo efflux via P-glycoprotein transporters, thus failing to elicit any positive outcomes in tauopathy animal models [68]. Similarly, in a number of experiments, it was only possible to measure the activity of the drug in terms of peripheral pharmacodynamics in PBMCs or CSF since direct measurement in astrocytes/microglia could not be accomplished [72,78].

In addition, sampling constraints severely compromised the validity and applicability of results. Several studies used small sample sizes of humans, hindering data analysis and making it difficult to conclusively assess factors like gender-specific signaling pathways [22,39,46]. Other experiments were conducted using only young males, leaving no room for discussion on necroptosis associated with age or gender differences in AD pathogenesis [33]. Likewise, unusual variant frequency rates in replication groups undermined independent statistical significance despite initial positive results [73]. Moreover, sampling constraints were present in kinetic modeling experiments in primates due to the small number of available subjects [31].

Further challenges in interpretation are presented by critical translational issues. For instance, reliance on animal models can be inappropriate due to inevitable marked differences between human and rodent neural biology, the function of the blood–brain barrier, and the immune system [34]. In addition, data gathered from diabetes models, among other conditions, do not necessarily correspond to the specific etiology of AD. Moreover, there is a chance that there was a certain selection bias when only genes highly expressed in neurons were studied genetically [73].

Finally, there were several technical and experimental limitations that blocked studies to show significant results. In some cases, it was not possible to reliably detect essential proteins involved in necroptosis like RIPK3 and p-MLKL in human brain tissue homogenates, probably due to low protein levels or insufficient antibodies [46]. Some experiments used just one RIPK1 inhibitor concentration and did not characterize any necroptosis activity independent from neurons, which made it difficult to draw a mechanistic conclusion [38]. Other results were still correlational rather than causal; for example, some research correlated the presence of iron deposits with changes related to necroptosis, and behavioral tests in mice were prone to bias due to motor function deficits [33]. Technical issues included an incomplete clinical dataset and PET imaging techniques’ limitations, including high costs, low accessibility, and ionizing radiation [31].

## 11. Knowledge Gaps

There have been promising developments in the field. However, many uncertainties remain that need to be further investigated. One of the biggest gaps in the field is the lack of definitive proof that the presence of RIPK1/RIPK3/MLKL in cells is the cause of cell death. RIP kinases have non-death roles (e.g., in inflammation, autophagy), and “necroptosis” markers can accumulate without simultaneous cell lysis because of late pathway checks [80,81]. In fact, it is suggested that increased levels of necrosome components in neurons can sometimes indicate death signals or new functions [39]. A new study on brain pathology has revealed that the necrosome complex does not accumulate in some regions such as the spinal cord and central cortex [82]; this could be explained by regional differences or technical reasons. Therefore, it remains unclear whether necroptosis is the primary cause of neuronal loss in sporadic Alzheimer’s or merely a side effect accompanying inflammation. Furthermore, it is not yet clear when RIPK1 is activated in AD.

Nevertheless, while evidence implicating RIPK1 in AD pathology continues to accumulate, there is still uncertainty regarding whether or not the activation of this pathway can be considered as the primary causative mechanism leading to neurodegeneration or a secondary response to the ongoing neurodegenerative insults and inflammatory processes. A number of experimental studies have shown that pharmacological and genetic inhibition of RIPK1 leads to improvement of neuroinflammatory processes, synaptic dysfunction, and cognitive impairment [18,21,35]. Regardless, a lot of evidence remains correlational in nature, with increased levels of RIPK1 activation being observed only as a reflection of the progressive state of AD. Furthermore, neuroinflammation itself may function as both a defensive reaction against the disease progression and an aggravating factor depending on the stage of the pathology development and cellular context. Thus, while early-stage activation of microglia helps to clear Aβ plaques from the brain and stimulates tissue repair, later on, it may become maladaptive and toxic for neurons. Therefore, despite the apparent role of RIPK1 in the development and progression of AD, it may not function as the primary cause of neurodegeneration but rather as its amplification. Longitudinal in vivo studies and cell-type-specific analyses will therefore be essential to determine whether RIPK1 activation precedes neurodegeneration or predominantly emerges as a secondary response during disease progression [15,83,84]. The number of studies supporting that vascular and inflammation pathologies and mechanisms contribute as the initiators of dementia is few. (e.g., white matter alterations, CSF TNF level elevation) [85], and there are no longitudinal in vivo studies. Whether the necroptosis mechanism plays an early or late role in the development of the disease or is involved in the whole progression of the disease is still unknown.

A key constraint in the understanding of RIPK1 signaling in AD includes the high prevalence of concurrent neurological pathologies in aged brains. Neuro-pathology studies show that multiple pathologies instead of just amyloid/tau pathology are common in older subjects with dementia. Pathologies such as cerebrovascular disease, TDP-43 pathology, hippocampal sclerosis, Lewy body pathology, and cerebral amyloid angiopathy are commonly associated with Alzheimer’s patients and can affect neuro-inflammatory and necroptotic signaling independently [86]. Significantly, most of these concomitant neuro-pathologies involve sustained activation of innate immunity, endothelial dysfunction, oxidative stress, and signaling involving TNF, among others, and therefore, they can directly impact RIPK1 signaling. Indeed, both vascular injury and ischemia have been implicated as inducers of RIPK1-dependent necroptosis, while α-synuclein/TDP-43 pathology also contributes to inflammatory RIPK1 signaling in animal models of neuro-degeneration [87]. In this regard, therefore, some of the increase in RIPK1 activity in AD brains can be accounted for by the presence of other neurological comorbid conditions in addition to specific AD pathogenesis. The importance of investigating the contribution of comorbid pathologies in RIPK1 activation needs to be examined further in future clinicopathological studies [84]. What is the contribution of neurons, glia, and endothelium to necroptosis-related brain damages is unknown, too. Genetic studies (e.g., SHARPIN mutations, RIPK1 single-nucleotide polymorphism) suggest that RIPK1 is a risk factor for Alzheimer’s disease; however, there are no large-scale genetic studies that have proved that RIPK1 is a genetic locus for Alzheimer’s disease. In terms of therapy, safety is another challenge as RIPK1 is an important kinase, and chronic treatment may affect the immune response or cell death signaling in other situations. Previous RIPK1 inhibitors (DNL104) caused liver toxicity and inhibited PK [42]. Recent industry failures to develop new drugs such as GSK2982772 and DNL747 showed that tolerability is another challenge [82]. Lastly, the extent to which RIPK1 inhibition improves cognition and the right patient population to use the drug candidate are still unknown.

## 12. Future Directions and Clinical Implications

There are a number of ways to take advantage of the potential role of RIPK1 in Alzheimer Disease (AD). Mechanistic studies could be performed to determine the appropriate triggers that lead to the activation of RIPK1 in the brain; for example, it could be useful to study how Aβ and tau interact with cytokine signaling to see if the cofactor Protein62/UV Radiation Resistance-Associated Gene (p62/UVRAG) performs a regulatory role in the control of RIPK1 expression in neurons [88].

Second, a variety of RIPK1 deletion experiments could be performed to quantify the specific functions associated with RIPK1 in a neuroinflammatory context in response to cell-specific models (in terms of the relative contribution of neurons versus microglia in experimental models of AD), and finally, necroptosis of non-neuronal cells such as astrocytes and pericytes remains an underexplored area. As an example of a potential new biomarker, plasma and CSF could be investigated to identify the levels of proteins associated with RIPK1, p-RIPK1, or MLKL and their cleaved forms. Therefore, it may also be beneficial to investigate the use of PET imaging to identify glial cells that are associated with necroptosis.

It should be assumed that any future drug candidates for use as inhibitors of RIPK1 will have to be both safe and able to cross the blood–brain barrier prior to clinical trial initiation. The failure of some promising drugs at early stages of testing highlights the necessity of selective inhibitors with appropriate efficacy and ability to penetrate the brain. While several studies have shown that the use of such drugs results in decreased tau and amyloid-β accumulation, the effect of such drugs on the inflammatory response and behavioral disturbances caused by tau protein has yet to be studied [89]. Therefore, combination trials (RIPK1 inhibitors + anti-amyloid) could potentially be very enlightening and helpful due to the synergy of this pathology. Finally, because of the location of RIPK1 in the inflammation pathway, there is great interest in the role played by this protein in neurodegenerative disorders. This is where the discussion centers on AD. The possible advantages of inhibiting RIPK1 in the context of AD include prevention of neuronal cell death through necroptosis and reduction in inflammation, which can result in preservation of BBB integrity and slower progression of cognitive impairment. Collectively, these facts indicate that RIPK1 is an important multi-faceted player in AD, and further research has the potential to capitalize on these findings soon.

## 13. Discussion

For many years, the AD research has been primarily focused on amyloid plaques and neurofibrillary tangles. Yet mounting evidence now makes clear that these two hallmarks alone cannot fully explain the progressive neuronal loss that drives the disease. Increasingly, attention has turned to necroptosis, with RIPK1 at its core, as a critical mechanism that not only explains aspects of cell death in AD but also links them to chronic inflammation and vascular disruption. Unlike apoptosis, which is not commonly found in AD brains, necroptosis is consistently marked by elevated RIPK1, RIPK3, and MLKL, suggesting that this pathway is deeply embedded in the degenerative process [4,7,19,32,35]. This recognition reframes AD not only as a disorder of protein aggregation but also as a disease fueled by regulated inflammatory cell death [5,21,22,37].

One of the most striking findings about RIPK1 is the way it brings together two notions that, for years, divided the AD field: the amyloid hypothesis and the tau hypothesis. Instead of seeing them as competing explanations, RIPK1 shows how they can be part of the same chain of events. Studies in both humans and experimental models suggest that when amyloid oligomers accumulate, they set off RIPK1, which then pushes neurons toward necroptosis and accelerates cognitive decline. When researchers block RIPK1 in animal models, this destructive process slows down, then neurons survive longer, the burden of pathology lightens, and memory performance is preserved. In other words, targeting RIPK1 doesn’t just reduce one hallmark or the other; it interrupts the pathogenic conversation between amyloid and tau, offering us a glimpse of how the disease might be disarmed at its core [4,7,16,18,19,23]. Tau pathology has a similar pathway: hyperphosphorylated tau aggregates are frequently observed along with necroptosis markers, and mutant tau species can directly trigger RIPK1-mediated cell death. Studies with human neurons transplanted into amyloid-rich brains have shown that amyloid worsens tau pathology by enhancing RIPK1 signaling through MEG3, creating a cycle in which the two hallmark lesions amplify one another. These findings point to RIPK1 as a link between amyloid and tau, suggesting that both converge on necroptotic signaling to drive neurodegeneration [7,16,18,19,23,46].

RIPK1 not only works on neurons but also strongly affects the support cells of the brain, including microglia and astrocytes. Through treatment with amyloid or tau, they release cytokines that trigger RIPK1, which can cause necroptosis and drive the cells to release their contents into the surrounding tissue. This initiates a vicious cycle of neuroinflammation. And on top of that, RIPK1 drives microglia towards a damaging, M1-like phenotype, and inhibition allows them to take on more of a useful function for clearing amyloid. In short, RIPK1 causes both neuronal harm and immune setting changes that continue to make chronic inflammation feed in AD. The fact that a single kinase can orchestrate such far-reaching effects suggests integral functions of RIPK1 to the complex ballet of neurodegeneration and inflammation that defines this disease.

In Alzheimer’s disease, the BBB, which normally serves as a protective barrier for the brain, gets compromised at an early stage in the disease course. Recent studies indicate that activation of RIPK1 in endothelial cells leads to such a compromise directly. Studies on post-mortem human brains have revealed that activation of RIPK1 in endothelial cells leads to impaired integrity of tight junctions and pericyte depletion, two critical components of healthy vessels. Based on animal studies, inhibition of RIPK1 may serve to preserve these vessel properties as well as prevent any leakage and cognitive impairment. Altogether, it may be stated that vascular pathology seen in Alzheimer’s is not separate from neuron or glia cell pathology in AD; instead, it forms a common crew. Thus, RIPK1 appears as a key player that links several features of this complex disorder. The translational importance of such findings is evident from genetic ablation and pharmacological inhibition studies showing reduced neuronal loss, lack of inflammation, synaptic preservation, and maintenance of vascular integrity in preclinical models.

Several inhibitors have been used in human trials. One of them, DNL747, engaged its target but was discontinued because of concerns related to safety issues. Fortunately, other molecules that were tested later, including DNL788, SIR-2446, and GFH312, exhibit better pharmacological profiles and brain penetrance, and their phase I clinical trial revealed no safety concerns. None of them, however, have proven to be therapeutically effective in AD patients, but it still provides hope that the therapeutic benefits of RIPK1 inhibition depend on finding the proper balance between safety, efficacy, and selectivity [18,33,38,68,72,76,78,79].

At present, there remain a number of unresolved issues. For example, in studies on tauopathies, blockade of RIPK1 decreases inflammation, but it is insufficient for preventing neuronal death; therefore, necroptosis belongs to the greater class of cellular deaths. This reservation concerning the inability of RIPK1 blockade alone to fully protect against neurodegeneration highlights the necessity of combination therapies using inhibitors and either antiamyloid or antitau medications. Another potential issue concerns safety because RIPK1 contributes greatly to immune defense; thus, chronic inhibition may impair the body’s capability to resist pathogens. Furthermore, the mere presence of necrosome components does not guarantee necroptosis because of sequestration of these proteins in a non-lethal manner, e.g., autophagy or inflammation. There are a number of other questions that have yet to be answered. Is necroptosis an early pathological phenomenon or rather a booster factor, and what populations of cells are involved in RIPK1-related pathology? While RIPK1 variants, such as SHARPIN, were linked to aberrant regulation of RIPK1 and the increased AD risk, there is currently not enough genetic evidence in comparison to humans [22,39,77,80,81,82,85].

Additionally, studies should be conducted to identify the mechanisms by which amyloid, tau, and inflammation converge at RIPK1 and the factors that mediate such convergence. Furthermore, studies should also explore the influence of comorbid diseases in modulating RIPK1 pathways and its contribution to neuroinflammation due to necroptosis in AD. Among the diseases associated with AD are cerebrovascular diseases, diabetes mellitus, obesity, hypertension, TBI, and systemic inflammation. These diseases contribute to oxidative stress, mitochondrial dysfunction, vascular dysregulation, and an increased level of TNF inflammatory pathway, which in turn could increase the activity of RIPK1 pathway and ultimately result in necroptosis. “Inflammaging,” an age-related process, could lower the threshold for chronic inflammatory responses mediated by RIPK1 pathways. Thus, RIPK1 activation in AD can be seen as the effect of the combined impact of amyloid and tau pathology and various metabolic, vascular, and inflammatory factors [45]. The use of neuron-, glia-, and endothelial cell-specific knockouts may provide clues about the contributions made by different types of cells. Possible translational advances include the development of biomarkers that monitor RIPK1 activation, phospho-RIPK1, RIPK3, or MLKL fragments in cerebrospinal fluid (CSF) or serum, or even imaging agents. Among the possible therapeutic strategies, the most promising appears to be combination therapies, which may include RIPK1 inhibitors together with current disease-modifying treatments.

Collectively, the available evidence points to a pivotal role for RIPK1 beyond merely being a signaling molecule. Indeed, it seems to be a nexus where amyloid, tau, inflammation, and vascular dysfunction intersect. As such, it can be seen both as a mediator of the pathogenesis of the disease and as a potential therapeutic target. While questions still linger about when it is activated, what types of cells it acts on, and how safe it is to block it, the existing evidence seems to strongly indicate that necroptosis induced by RIPK1 plays a crucial role in AD pathogenesis. Given the intense focus of research into the biology of this molecule similar to amyloid and tau, it may indeed be possible to uncover new avenues for therapy in AD [9,18,52,68,72,77,79].

## 14. Conclusions

As a progressive and neurodegenerative disorder with complex heterogeneous etiology, AD arises from and develops through multiple pathological pathways, including Aβ deposition, hyperphosphorylation of tau, chronic inflammation, and vascular pathology. RIPK1 is at the intersection of all these pathways, not only initiating necroptosis but also dictating the immune response. In addition to contributing to neuronal death, RIPK1 has significant effects on microglial activity, the integrity of the BBB, and chronic inflammation, placing RIPK1 centrally in the disease process. Preclinical studies have brought some optimism, as they led to the demonstration that RIPK1 inhibition can reduce inflammation, protect neurons, and at least improve cognition in model systems. Translating the biomarker to clinical use, however, has met with challenges. For initial RIPK1 inhibitors, while effectively blocking inflammation, the relatively poor blood–brain barrier penetration and a restrictive safety profile precluded their use in clinical development. These agents are still being evaluated, but newer agents that degrade RIPK1 proteins have also been developed. Further studies are warranted to explore how RIPK1 operates in various brain cell types and to assess, inhibit, or degrade RIPK1 throughout disease progression. While collective evidence suggests that RIPK1 is a plausible drug target, it remains unexplored as to whether RIPK1 inhibition will be most effective alone or in combination with other approaches to become potential disease-modifying therapies.

## Figures and Tables

**Table 1:** Key preclinical and clinical evidence supporting RIPK1 involvement in AD and related neurodegenerative disorders. Created by the authors.

Aspect	Evidence	Models/Subjects	Key Findings	References
Biomarker Potential	RIPK1 and MLKL elevated postmortem	AD patient brains	↑ RIPK1/MLKL correlated with ↓ cognitive scores	[22,31,46]
	RIPK1 inversely correlates with brain weight	Human autopsy	Necroptosis burden reflects disease severity	[3]
	Necroptosis markers linked to behavioral deficits	APP/PS1 mice	Necroptosis severity parallels memory/behavioral impairment	[33]
	Oral RIPK1 inhibitor (DNL747) reduces p-RIPK1S166	AD patient PBMCs	Demonstrates on-target peripheral biomarker engagement	[72]
	Genetic susceptibility: SHARPIN variant	Human genetics	SHARPIN variant alters NF-κB regulation, ↑ risk	[73]
Therapeutic Strategies	Genetic inactivation of RIPK1 (Ripk1D138N), RIPK3 KO, MLKL KO	APP/PS1 mice	Protection against synaptic loss & memory impairment	[33]
	Nec-1 and Nec-1s	APP/PS1 mice	↓ neuroinflammation, ↓ neuronal loss, ↓ BBB leakage	[38]
	Repurposed kinase inhibitors (ponatinib, dabrafenib, sorafenib)	AD xenograft mouse model	Suppress RIPK3 activity, reduce pathology	[39]
	GFH312 (NCT04676711)	Phase II (healthy volunteers)	↓ p-RIPK1 in PBMCs postdose, blocks necroptosis. Discontinued due to adjusted strategy	[76]
	GSK2982772 (RIPK1 inhibitor)	Tauopathy models (FTD, PSP, AD patients & mice)	↓ Astrocyte reactivity but no prevention of hippocampal neurodegeneration, discontinued due to low efficacy.	[68]
	DNL104	Phase I (healthy volunteers)	Inhibited RIPK1 phosphorylation & CNS-Penetrant; discontinued due to postdose liver toxicity	[77]
	DNL747 (SAR443060)	Early Phase I, AD patients	Safe, CNS-penetrant, ↓ p-RIPK1 in PBMCs; discontinued due to toxicology + D11:D14	[72]
	DNL788 (SAR443820)	Phase I (healthy volunteers), Phase II (ALS, MS)	Safe, improved PK, FDA Fast Track in ALS; exploratory study in AD	[78]
	SIR-2446 (Sironax)	Phase I, AD & MS patients	Oral, CNS-penetrant, safe, potent target inhibition in biomarkers	[79]
Potential Research Areas	Biomarker validation	Human & mouse	A potential probe to “see” RIPK1 in the brain of humans require clinical research	[34,72]
	Disease model evaluation for therapeutic efficacy	APP/PS1 mice, tauopathy mice	RIPK1 inhibition reduces inflammation but, when used alone, does not prevent neuronal loss. Additionally, mouse models only partially reflect AD pathology	[33,68]
	Clinical translation	AD trials (DNL747, DNL788)	Safety/toxicity issues, limited CNS penetration remain barriers	[72,78]

Abbreviations: RIPK1, receptor-interacting protein kinase 1; MLKL, mixed lineage kinase domain-like protein; AD, Alzheimer’s disease; APP, amyloid protein precursor; PS1, presenilin 1; PBMCs, peripheral blood mononuclear cells; SHARPIN, SHANK-associated RH domain interactor; NF-κB, nuclear factor kappa B; RIPK3, receptor-interacting protein kinase 3; KO, knockout; Nec-1, necrostatin-1; Nec-1s, necrostatin-1 stable analog; BBB, blood–brain barrier; p-RIPK1, phosphorylated RIPK1; FTD, frontotemporal dementia; PSP, progressive supranuclear palsy; CNS, central nervous system; ALS, amyotrophic lateral sclerosis; MS, multiple sclerosis; PK, pharmacokinetics.

**Table 2:** Major limitations and setbacks in RIPK1-targeted preclinical and clinical studies in AD and related neurodegenerative disorders. Created by the authors.

Limitation Category	Key Limitation/Setback	References
Discontinuation and Safety Issues/Side Effects	Reports the failure of the Phase 2 HIMALAYA trial for ALS; DNL788 did not meet the primary endpoint for improved functional outcomes.	[39]
	GFH312 was withdrawn due to an adjusted clinical development strategy by the sponsor.	[76]
	Discontinued due to long-term nonclinical toxicology findings in monkeys, specifically immune-mediated anemia and thrombocytopenia.	[72]
	DNL104 was discontinued due to liver function abnormalities (elevated ALT/AST) in healthy volunteers.	[78]
	Dosing halted in the 400 mg multiple-dose cohort by the sponsor due to a high frequency of rash morbilliform.	[79]
Brain Penetrance and Target Site Issues	Target engagement could not be measured directly at human CNS target sites (astrocytes and microglia); results were limited to peripheral blood and CSF.	[72]
	GSK2982772 suffers from compromised brain penetrance due to efflux by P-glycoprotein; it showed no beneficial effect on neurodegeneration in tauopathy models.	[68]
	Study limited by a short treatment duration (14 days) and PD assessments restricted to the peripheral level in human PBMCs.	[78]
Sampling Limitations	Similar technical and sample size limitations applied; inability to reach definitive proof for specific gender differences in pathway activation.	[22,39,46]
	Relatively small human sample size (63 cases), making it difficult to statistically analyze linear model terms with more than three variables.	[46]
	Modest initial discovery cohort (202 patients); replication cohort carriers were too rare to reach independent statistical significance.	[73]
	Focused exclusively on young male animals, preventing any conclusions about necroptosis in the aged or female brain.	[33]
Technical and Experimental Constraints	Datasets for human variables like brain weight and MMSE were incomplete for the full cohort.	[3]
	General PET imaging limitations including high cost, limited availability, and radiation exposure.	[31]
	Technical failure to detect key proteins (RIPK3, p-MLKL) in human brain homogenates via Western blot	[46]
	The study evaluated only a single drug (Nec-1) at a single dose level; distribution of necroptosis beyond neurons was not identified.	[38]
	Demonstrated only a spatial correlation but not a causal link for iron deposition; mouse memory tests are easily confounded by motor dysfunction.	[33]
Statistical Robustness	Primate kinetic modeling had low statistical power due to a very small number of animals. Rodent studies were limited to males.	[31]
Translational Gaps	Heavy reliance on rodent models (brain structure and BBB differences). Findings in diabetic models may not translate to AD.	[34]
Selection Bias	Potential risk gene selection bias because the filtering focused only on genes expressed in the brain	[73]

Abbreviations: AD, Alzheimer’s disease; ALS, amyotrophic lateral sclerosis; ALT, alanine aminotransferase; AST, aspartate aminotransferase; BBB, blood–brain barrier; CNS, central nervous system; CSF, cerebrospinal fluid; MMSE, Mini-Mental State Examination; Nec-1, necrostatin-1; PBMCs, peripheral blood mononuclear cells; PD, pharmacodynamic; PET, positron emission tomography; p-MLKL, phosphorylated mixed lineage kinase domain-like protein; RIPK1, receptor-interacting protein kinase 1; RIPK3, receptor-interacting protein kinase 3.

## Data Availability

No new data were created or analyzed in this study.

## Abbreviations

The following abbreviations are used in this manuscript:

Aβ	Amyloid β
AD	Alzheimer’s disease
ALS	Amyotrophic lateral sclerosis
APOE	Apolipoprotein E
APP	Amyloid protein precursor
BBB	Blood–brain barrier
Caspase-8	Cysteinyl aspartate-specific protease 8
CCL	C–C motif chemokine ligand
cIAP1/2	Cellular inhibitor of apoptosis protein 1 and 2
CNS	Central nervous system
CSF	Cerebrospinal fluid
CSF-2	Granulocyte–macrophage colony-stimulating factor (GM-CSF)
CXCL	C–X–C motif chemokine ligand
DAMPs	Damage-associated molecular patterns
FADD	Fas-associated protein with death domain
GVD	Granulovacuolar degeneration
IFN	Interferon
IL	Interleukin
KO	Knockout
LUBAC	Linear ubiquitin chain assembly
MEG3	Maternally expressed gene 3 (long non-coding RNA)
MIF	Macrophage migration inhibitory factor
MLKL	Mixed lineage kinase domain-like protein
MS	Multiple sclerosis
mtDNA	Mitochondrial DNA
NaFl	Sodium fluorescein
Nec-1	Necrostatin-1
Nec-1s	Necrostatin-1 stable analog
NF-κB	Nuclear factor kappa B
p62/UVRAG	Protein 62/UV Radiation Resistance-Associated Gene
p-MLKL	Phosphorylated MLKL
p-RIPK1	Phosphorylated RIPK1
p-RIPK3	Phosphorylated RIPK3
PBMCs	Peripheral blood mononuclear cells
PET	Positron emission tomography
PK	Pharmacokinetics
PS1	Presenilin-1
PSP	Progressive supranuclear palsy
pTau	Phosphorylated tau
PTM	Post-translational modification
RHIM	Receptor-interacting protein homotypic interaction motif
RIPK1	Receptor-interacting protein kinase 1
RIPK3	Receptor-interacting protein kinase 3
ROS	Reactive oxygen species
SHARPIN	SHANK-associated RH domain interactor
TAK1	Transforming growth factor beta-activated kinase 1
TDP-43	TAR DNA-binding protein 43
TEER	Transepithelial electrical resistance
TLR4	Toll-like receptor 4
TNF-α	Tumor necrosis factor alpha
TNFR1	Tumor necrosis factor receptor 1
TNFSF10	Tumor necrosis factor-related apoptosis-inducing ligand (TRAIL)
TRAF2/5	Tumor necrosis factor-associated factor 2 and 5
UVRAG	UV radiation resistance-associated gene
zVAD-fmk	Benzyloxycarbonyl-Val-Ala-Asp-fluoromethylketone
